# Obituary

**Published:** 1912-01-27

**Authors:** 


### Obituary.

Dr. Alfred Wolff, of Holland Park, has died at Birmingham in his 59th year. He qualified from St. Thomas's Hospital in 1876.

Dr. James Kershaw Milne, of Merton Park, Surrey, has died at the age of 56. He received his medical training at Owen's College, Manchester, and qualified at the Royal College of Surgeons in 1876. He was formerly house physician at the Manchester Royal Infirmary, and also held a resident appointment at the Monsall Fever Hospital.

Dr. Gordon Moir, surgeon in the Royal Navy, has died at the early age of 33, from an accidental overdose of morphia. He was a student of Guy's Hospital, where he qualified in 1903 at the Conjoint Board. He held a resident appointment at the Hertford General Infirmary, and then entered the Naval Medical Service. He had contributed papers on clinical subjects to the medical Press.

Dr. Herbert Robert Power, of Isleworth, has died at a comparative early age. He was formerly House Surgeon and Resident Casualty Officer at St. Mary's Hospital, from which he qualified in 1895 at the Conjoint Board. For some time he acted as District and Sanitary Medical Officer for Kurnool, Madras, and then settled in Isleworth, where he held the appointment of Medical Officer to the Mogden Isolation Hospital. Dr. Power was also a surgeon in the Royal Naval Volunteer Reserve.

Dr. Cecil Yates Biss, F.R.C.P., late physician to the Brompton Hospital for Consumption, has died at his residence in St. John's Wood, where he had been living in retirement. Dr. Biss was formerly physician to outpatients at Middlesex Hospital, and lecturer on pharmacology to the Medical School, besides holding many other appointments to well-known charities. His contributions on clinical subjects were very numerous, and he was one of the examiners to the Apothecaries Society and the Royal College of Physicians. Dr. Biss started his successful career as a scholar of Downing College, Cambridge, where he obtained a first class in the Natural Sciences Tripos of 1875. He qualified in 1880, and was elected a Fellow of the Royal College of Physicians in 1889. He was a student at St. Bartholomew's Hospital.

